# Genetics of causal relationships between circulating inflammatory proteins and postherpetic neuralgia: a bidirectional Mendelian randomization study

**DOI:** 10.3389/fneur.2024.1405694

**Published:** 2024-06-21

**Authors:** WenHui Liu, HuiMin Hu, Chen Li, YiFan Li, Peng Mao, BiFa Fan

**Affiliations:** ^1^Graduate School, Beijing University of Chinese Medicine, Beijing, China; ^2^Department of Pain Management, China-Japan Friendship Hospital, Beijing, China

**Keywords:** inflammatory mediators, postherpetic neuralgia, genetic prediction, Mendelian randomization, genome-wide association studies

## Abstract

**Objective:**

According to data from several observational studies, there is a strong association between circulating inflammatory cytokines and postherpetic neuralgia (PHN), but it is not clear whether this association is causal or confounding; therefore, the main aim of the present study was to analyze whether circulating inflammatory proteins have a bidirectional relationship with PHN at the genetic inheritance level using a Mendelian randomization (MR) study.

**Methods:**

The Genome-Wide Association Study (GWAS) database was used for our analysis. We gathered data on inflammation-related genetic variation from three GWASs of human cytokines. These proteins included 91 circulating inflammatory proteins, tumor necrosis factor-alpha (TNF-α), macrophage inflammatory protein 1b (MIP-1b), and CXC chemokine 13 (CXCL13). The PHN dataset was obtained from the FinnGen biobank analysis round 5, and consisted of 1,413 cases and 275,212 controls. We conducted a two-sample bidirectional MR study using the TwoSampleMR and MRPRESSO R packages (version R.4.3.1). Our main analytical method was inverse variance weighting (IVW), and we performed sensitivity analyses to assess heterogeneity and pleiotropy, as well as the potential influence of individual SNPs, to validate our findings.

**Results:**

According to our forward analysis, five circulating inflammatory proteins were causally associated with the development of PHN: interleukin (IL)-18 was positively associated with PHN, and IL-13, fibroblast growth factor 19 (FGF-19), MIP-1b, and stem cell growth factor (SCF) showed reverse causality with PHN. Conversely, we found that PHN was closely associated with 12 inflammatory cytokines, but no significant correlation was found among the other inflammatory factors. Among them, only IL-18 had a bidirectional causal relationship with PHN.

**Conclusion:**

Our research advances the current understanding of the role of certain inflammatory biomarker pathways in the development of PHN. Additional verification is required to evaluate the viability of these proteins as targeted inflammatory factors for PHN-based treatments.

## 1 Introduction

PHN is a pain that persists for more than 3 months following the onset of herpes zoster virus (HZ) and is characterized by burning, tingling or stabbing sensations, accompanied by spontaneous pain, touch-induced pain and nociceptive hypersensitivity (1–4). The pathophysiology of PHN has yet to be fully elucidated, but it is primarily attributed to the latent presence of the varicella-zoster virus (VZV) in the dorsal root ganglion following initial infection (5, 6). A reduction in specific cellular immunity enables VZV to reactivate and replicate. Age and extent of lesions are significant factors associated with a high incidence, and the risk of reoccurrence is equally high as the risk of first occurrence (7–9). Neuroplasticity is responsible for the pathogenesis of PHN. The mechanisms involved may include peripheral sensitization, central sensitization, inflammatory responses, dysfunction of downstream regulatory systems, and deafferentation phenomena. Studies have indicated that there is a robust correlation between the emergence of PHN and immune T cells, inflammatory factors, and activation of microglia (10–12). PHN has a multifactorial pathogenesis and limited treatment options. Patients often display symptoms of anxiety and depression and, in severe cases, may engage in self-harm and suicidal behavior. Such behavior not only detrimentally affects patients’ quality of life but also imposes a significant economic burden. Addressing this challenge is of urgent importance (13).

The role of the inflammatory response in the pathogenesis of PHN is considered significant. The replication of latent VZV in sensory ganglia increases after reactivation, and new viral particles and released products can be transported via axons to the corresponding spinal cord segments and cerebral nociceptors, as well as to peripheral regions innervated by sensory neurons. This process triggers the production of inflammatory cells and immune cell infiltration, causing inflammatory reactions, hemorrhagic necrosis, neuronal loss and demyelination in the skin, dorsal root ganglia and peripheral nerves. These changes contribute to nerve remodeling and ultimately result in chronic neuropathic pain (NP) (14–16). Simultaneous secondary inflammatory responses may result in lowered thresholds and increased sensitivity of peripheral nerve injury receptors, thus promoting peripheral sensitization (17–19).

Moreover, relevant studies have shown that pro- and anti-inflammatory cytokines are strongly linked to NP and other chronic pain conditions in humans. There is also substantial evidence for the analgesic effects of anti-inflammatory cytokines in animal models (20, 21). Recent studies on inflammatory cytokines, HZ and PHN have shown that the higher the expression of the proinflammatory factors TNF-α, IL-6, and IL-18 is, the more severe the inflammatory response induced, resulting in more severe irreversible nerve damage and longer-lasting chronic pain symptoms (22–24). The anti-inflammatory factors IL-4 and IL-10 have also been reported to be involved in the pathogenesis of PHN, and whether the anti-inflammatory factor IL-13 is related to this process has yet to be fully elucidated (25, 26). Chemokine levels may also be important, with Kensuke Fukuchi’s research indicating a crucial role for CXCL13 in generating herpes zoster-specific antibodies (27, 28). However, the precise pathogenesis and inflammatory mechanisms of action have yet to be fully elucidated and require ongoing exploration.

GWASs test millions of genetic variants in the genomes of many individuals to identify genotype–phenotype associations and have revolutionized the field of complex disease genetics over the past decade, providing a powerful approach to studying the genetic basis of complex diseases. Traditional observational studies are susceptible to confounding and bias. In contrast, MR is a statistical method based on GWAS data and Mendel’s law of free association to determine the causal relationship between exposure and disease, which can effectively avoid potential environmental confounding by exploiting the random assignment nature of genetic variants. Furthermore, it avoids reverse causality by having a reasonable causal time sequence between exposure and outcome. This approach is therefore more robust and reliable. The two-sample MR analysis adopted in this study allows for the association of SNP exposures and SNP results from independent GWAS, and the use of genetic variation as a proxy variable for exposures to infer causal associations between exposures and disease and combine them into a single causal estimate by comparing differences in disease incidence among individuals with different genetic variants (29–31). Therefore, this study implemented MR analysis to determine the bidirectional complex causal relationship between 94 circulating inflammatory proteins and PHN, explaining its pathogenesis and opening new avenues for targeted clinical treatment.

## 2 Materials and methods

### 2.1 Study design and data sources

**Figure 1:**
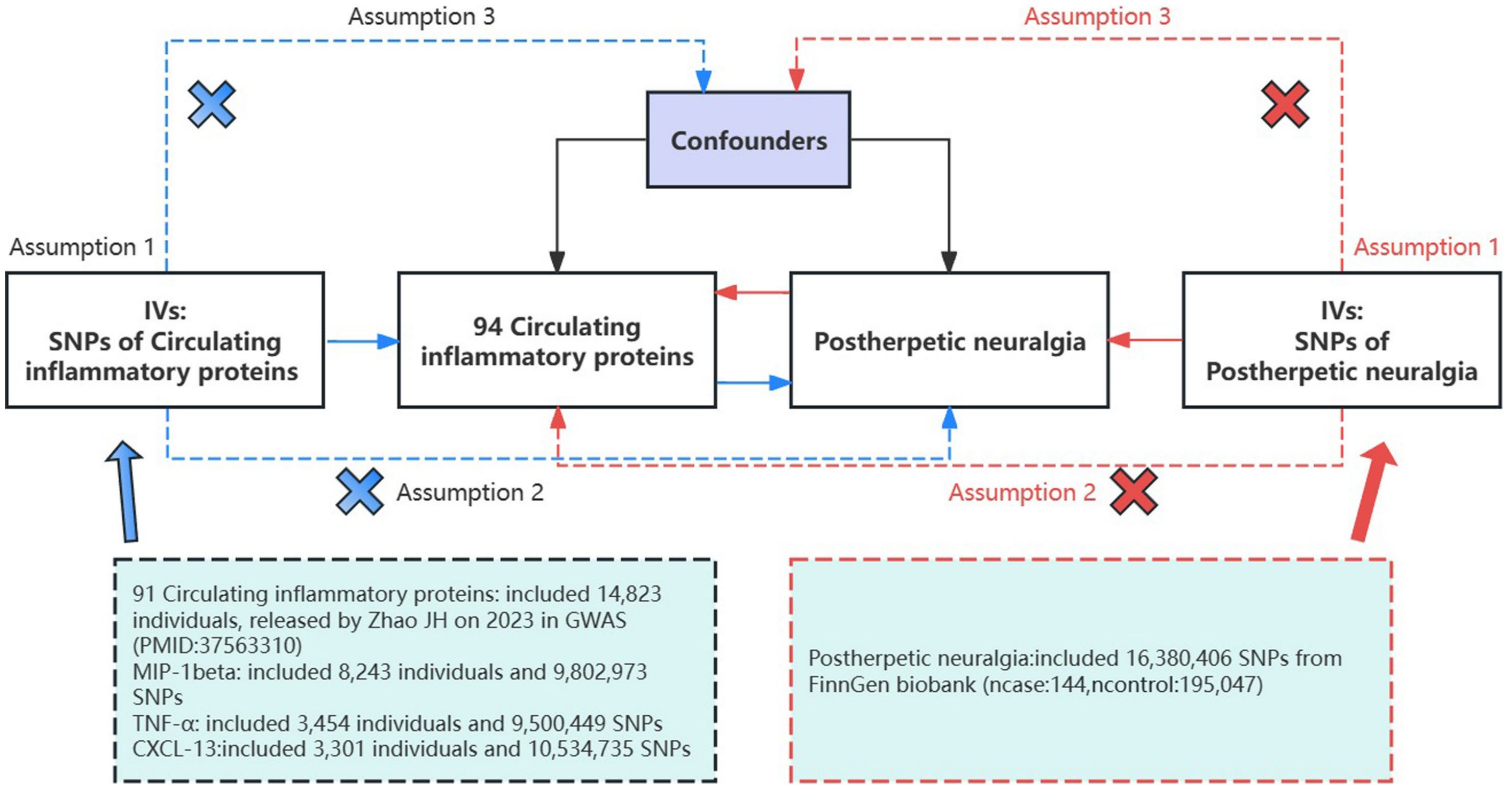
Assumptions and study design of a bidirectional MR study of the association of 94 circulating inflammatory proteins with PHN; The blue straight line represents the forward analysis, i.e., circulating proteins are the exposure and PHN is the outcome; the red straight line represents the reverse analysis, i.e., PHN is the outcome and circulating proteins are the exposure. GWAS, genome-wide association study; IVs, instrumental variables; SNPs, single-nucleotide polymorphisms.

Based on the GWAS of circulating inflammatory proteins and PHN, this study screened eligible instrumental variables (IVs) for MR analysis to explore the bidirectional causality between them. This study strictly followed the three assumptions of MR analysis: (1) relevance: the selected IVs were correlated with exposure; (2) independence: the IVs were not associated with potential confounders; and (3) exclusivity: the IVs affected the outcome only through the target exposure pathway (32) (Figure 1). As all the data used in this study were publicly available, no ethical clearance was needed.

GWAS datasets for 91 circulating inflammatory proteins were obtained from the most comprehensive meta-analysis published by Zhao JH in 2023, with a cumulative participant count of up to 14,823 individuals (33). The datasets for MIP-1b and TNF-α were both released by Ahola-Olli AV in 2016. The MIP-1b dataset originates from ebi-a-GCST004433 and comprises 8,243 samples, containing 9,802,973 single nucleotide polymorphisms (SNPs). Moreover, the TNF-α dataset was sourced from ebi-a-GCST004426 and consisted of 3,454 individuals with a total of 9,500,449 SNPs (34). The CXCL-13 dataset from prot-a-743 contained 3,301 samples with 10,534,735 SNPs (35). All participants in the dataset on inflammatory genetic variants were of European origin and provided informed consent. The PHN dataset was obtained from finn-b-G6_POSTZOST (FinnGen biobank analysis round 5), which was also from a European population, with a sample size of 144 and a control group of 195,047 individuals and 16,380,406 SNPs (36).

### 2.2 Selection of instrumental variables

The selected IVs should fulfill the three assumptions of MR analysis and guarantee the robustness and reliability of the MR analysis. (1) relevance: Initially, we acquired circulating inflammatory protein SNPs as IVs for forward analysis. Among these, the IVs of stem cell growth factor (SCF) and natural killer cell receptor 2B4 (NKR2B4) were thresholded (*p* < 5 × 10^−8^), whereas the other 92 exposures and PHN were examined using a threshold (*p* < 5 × 10^−6^) owing to the limited number of SNPs collected at usual thresholds. Subsequently, SNPs that met the threshold (*p* < 5 × 10^−6^) were used as IVs for PHN in the reverse analysis, the thresholds for inflammatory factors remained unchanged from the forward analysis. Moreover, to ensure a strong association between IVs and exposure, we calculated the F statistic for IVs. Any SNP with an *F* value lower than 10 was considered a weakly biased instrumental variable and was excluded from the analysis. The F value was calculated using the formula F = β^2^/se^2^ or F = (N- k-1)/k × R^2^/(1-R^2^). (2) independence: The linkage disequilibrium (LD) between SNPs was then removed, as strong LD leads to bias (*r*^2^ < 0.001, clumping distance = 10,000 kb). (3): exclusivity: we used PhenoScanner data to eliminate potential confounders (37–39).^1^

### 2.3 Statistical analysis and sensitivity analyses

Following the identification of relevant SNPs and the removal of palindromic SNPs, we utilized the TwoSampleMR (37) and MRPRESSO (40) packages within R software (version R.4.3.1) to conduct a bidirectional MR analysis of 94 circulating inflammatory proteins with PHN. Five distinct methodologies were employed. The IVW method, which is advantageous in reducing confounding bias, enhancing statistical validity, flexibility, ease of interpretation, and scalability, was selected as the primary approach for statistical exposure and outcome causality. This method integrates information from IVs and weights the analysis by using the inverse of their variance as a weight. The MR Egger, the weighted median, the simple model, and the weighted model were identified as the four auxiliary methods. *p* < 0.05 indicated statistical significance for the IVW method; i.e., exposure was associated with the outcome (36, 39, 41). In order to test for multivariate validity and reduce the bias in the MR estimation, the *p*-value of the global test for MR-PRESSO and the p-value of the intercept obtained from the MR Egger regression were used. Cochran’s Q statistic (MR-IVW) and Rucker’s Q statistic (MR Egger) were utilized to identify heterogeneity in the MR analyses, where *p* > 0.05 indicated a lack of heterogeneity in the IVs (32, 42). Leave-one-out sensitivity analysis was used to detect the effect of individual SNPs on the analysis results, and MR-PRESSO was also used to eliminate SNPs that cause bias. Additionally, we utilized visual aids such as forest plots and scatter plots for analysis purposes. Causality was deemed significant if the following three conditions were met: (1) IVW *p*-value <0.05, (2) No potential presence of pleiotropy or heterogeneity, (3) the estimates from the IVW, and MR-Egger methods were consistent in direction.

## 3 Results

### 3.1 Causal effects of circulating inflammatory proteins on PHN risk

**Figure 2:**
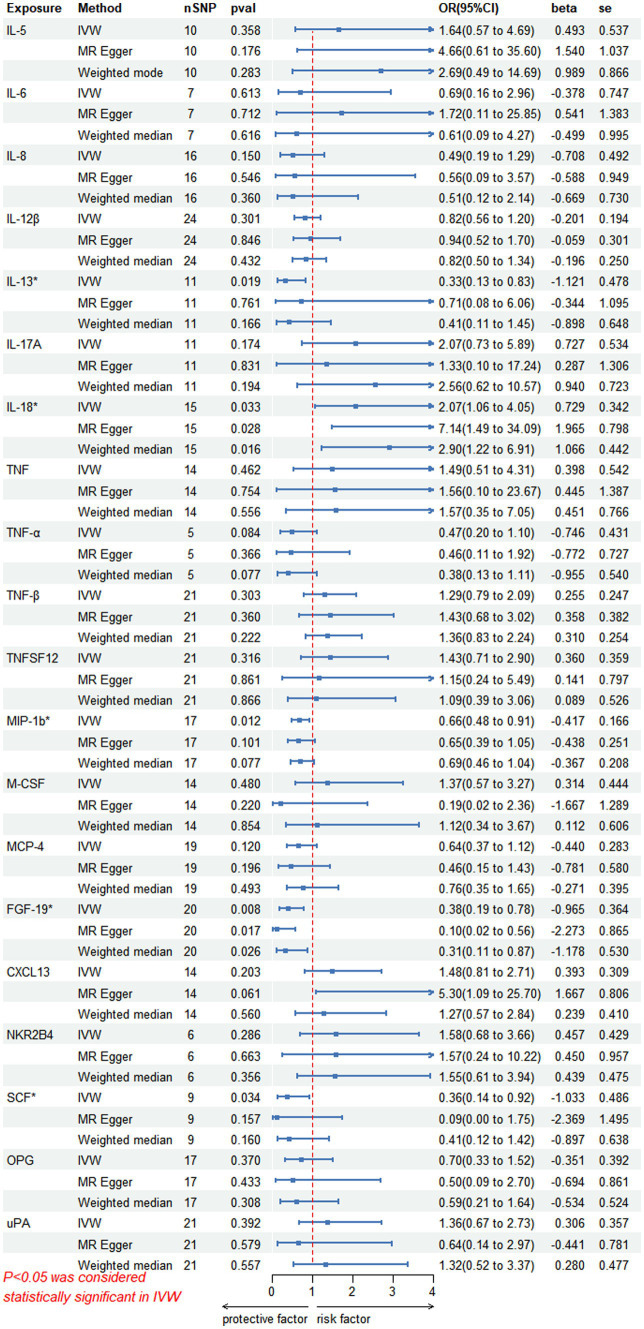
This forward MR using Circulating inflammatory proteins as exposure and PHN risk as outcome. The results of the IVW model demonstrated a significant correlation with a *p*-value lower than 0.05, consistent with genetic predictions. IVW, inverse variance weighted; OR, odds ratio; SNPs, single-nucleotide polymorphisms.

**Figure 3:**
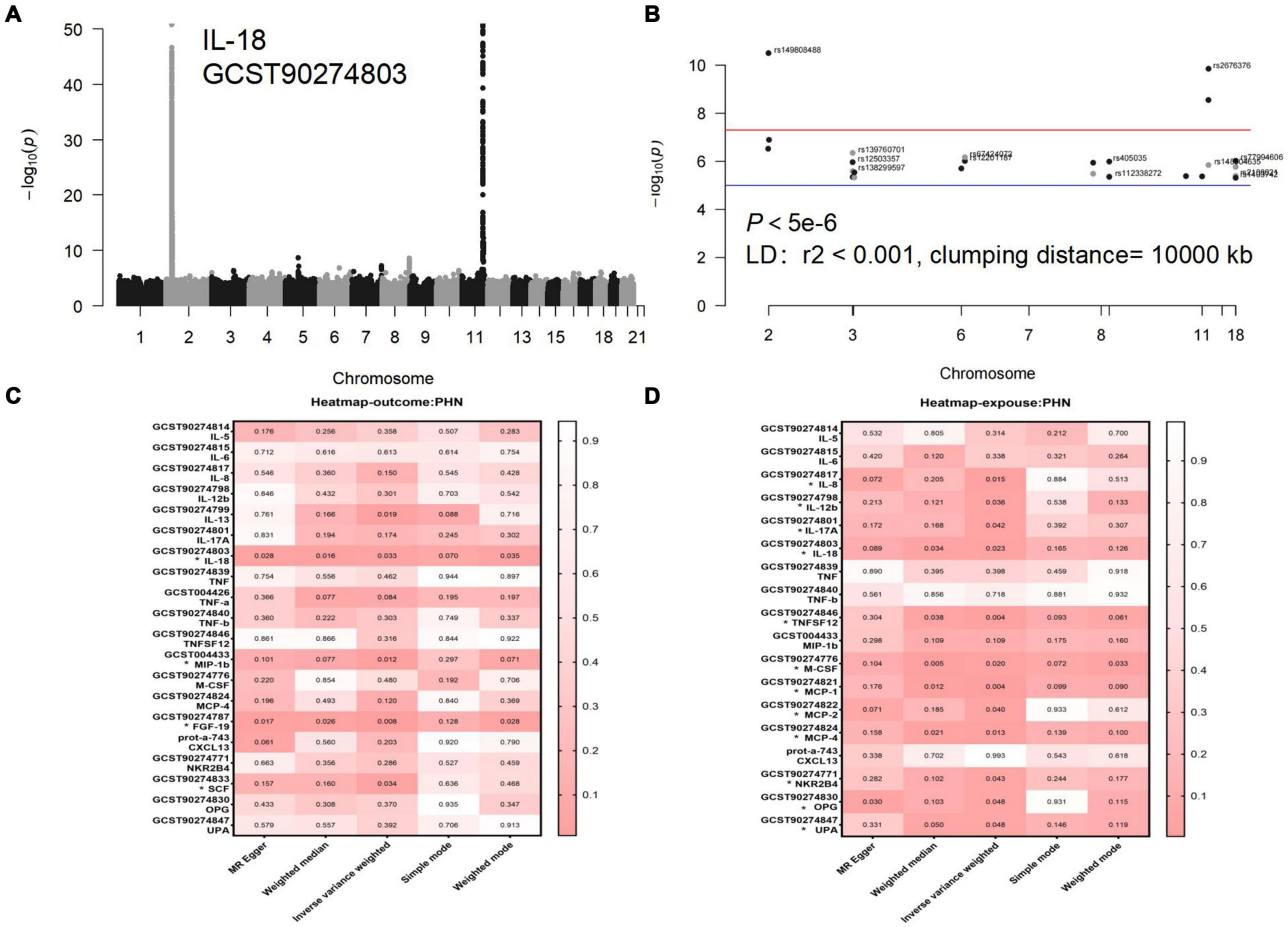
**(A)** Manhattan diagram for IL-18: The horizontal axis is the chromosome on which the SNP locus is located, and the vertical axis is the negative logarithm of the p value taken for each SNP calculation treatment. **(B)** Manhattan plot of IL-18 after selection of SNPS with a threshold less than 5e-6 and removal of LD. **(C)** Forward analyses. **(D)** Reverse analyses. The rightmost side of the heatmap shows a dendrogram based on Pval values between 0 and 1, with colors fading from red to white, with redder heatmap colors indicating stronger causality. Each row of the heatmap shows the GWAS number of the data and its corresponding circulating inflammatory protein name, with positive results marked with an *. Each column of the heat map is the 5 methods of the study, with the primary study method IVW in the center.

On forward MR analysis, we found that IL-18 (*p* = 0.033, 95% CI = 2.07 [1.06–4.05]) was causally associated with PHN, while MIP-1b (*p* = 0.012, 95% CI = 0.66 [0.48–0.91]), FGF-19 (*p* = 0.008, 95% CI = 0.38 [0.19–0.78]), IL-13 (*p* = 0.019, 95% CI = 0.33 [0.13–0.83]) and SCF (*p* = 0.034, 95% CI = 0.36 [0.14–0.92]) were inversely correlated with PHN (Figure 2). Based on the results of the forward analysis, we plotted a correlation heat map and forest plot to visualize our study (Figures 2, 3C).

**Figure 4:**
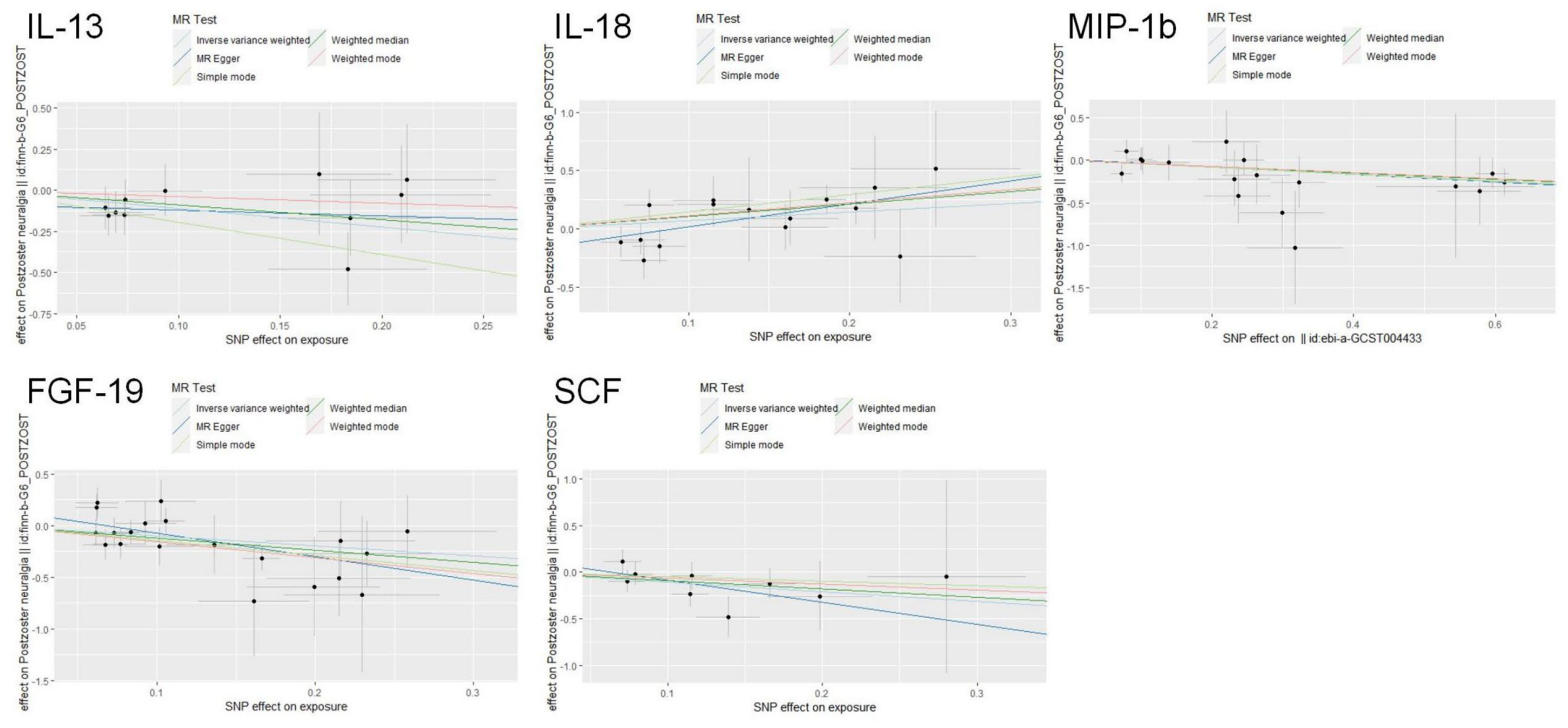
Scatter plots for the causal associations between 5 circulating inflammatory proteins and PHN.

**Figure 5:**
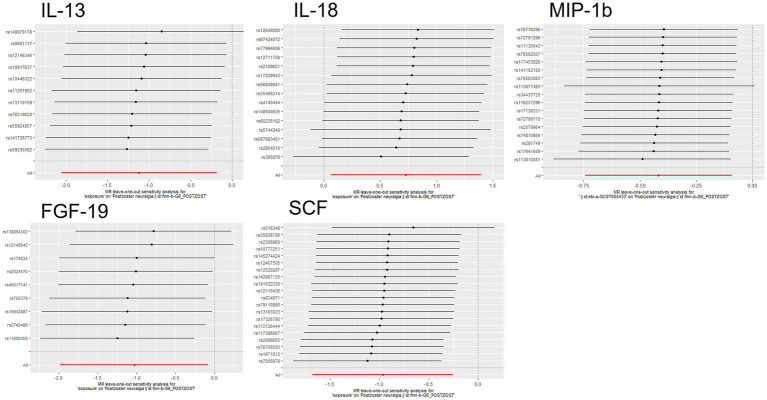
Forward MR leave-one-out plot for the causal association between 5 circulating inflammatory proteins and PHN. The vertical axis shows the SNPs included in the analysis.

The scatter plots showed relatively consistent trends across the different MR tests, with only IL-18 showing an upward trend as a risk factor (Figure 4). In sensitivity analyses, the results of leave-one-out analyses proved that the positive MR was reliable. Leave-one-out sensitivity analyses were performed for each of the SNPs to determine the causal effect of the five inflammatory factors on PHN. We found that the results were on the side of the zero line regardless of the exclusion of any of the SNPs (Figure 5). Genetic pleiotropy did not bias the results according to the MR-Egger regression intercept method.MR-PRESSO analyses demonstrated that horizontal pleiotropy did not exist in the MR studies (*p* > 0.05) and Cochran’s Q test did not show significant heterogeneity (*p* > 0.05). Table 1 However, no causal relationships were found between the remaining 89 circulating inflammatory proteins and PHN.

**Table 1:** Heterogeneity and pleiotropy analyses of the causal effect of circulating inflammatory protein levels on PHN.

Exposure	Heterogeneity test				Pleiotropy test	
	IVW Q	*P*	MR-Egger Q	*P*	MR-Egger *P*	PRESSO *P*
IL-5	8.247	0.509	6.855	0.552	0.271	0.512
IL-6	3.952	0.683	3.329	0.649	0.465	0.743
IL-8	10.468	0.789	10.446	0.728	0.883	0.779
IL-12β	22.261	0.504	21.880	0.467	0.543	0.608
IL-13	4.949	0.894	4.326	0.888	0.450	0.900
IL-17A	6.723	0.751	6.587	0.680	0.721	0.764
IL-18	13.875	0.459	10.931	0.616	0.109	0.494
TNF	11.605	0.560	11.603	0.478	0.971	0.578
TNFα	1.627	0.803	1.625	0.653	0.967	0.831
TNFβ	27.048	0.133	26.864	0.107	0.722	0.241
TNFSF12	20.796	0.409	20.692	0.354	0.760	0.421
MIP-1b	8.366	0.937	8.354	0.908	0.912	0.373
M-CSF	9.376	0.743	6.700	0.876	0.127	0.749
MCP-4	13.001	0.791	12.547	0.765	0.509	0.789
FGF-19	17.714	0.541	14.935	0.666	0.112	0.517
CXCL13	14.236	0.357	11.350	0.499	0.115	0.8600
NKR2B4	1.029	0.960	1.029	0.905	0.993	0.964
SCF	6.134	0.632	5.240	0.630	0.376	0.656
OPG	11.190	0.797	10.990	0.753	0.661	0.823
uPA	3.257	0.204	23.498	0.216	0.295	0.209

### 3.2 Causal effects of PHN on circulating inflammatory proteins

**Figure 6:**
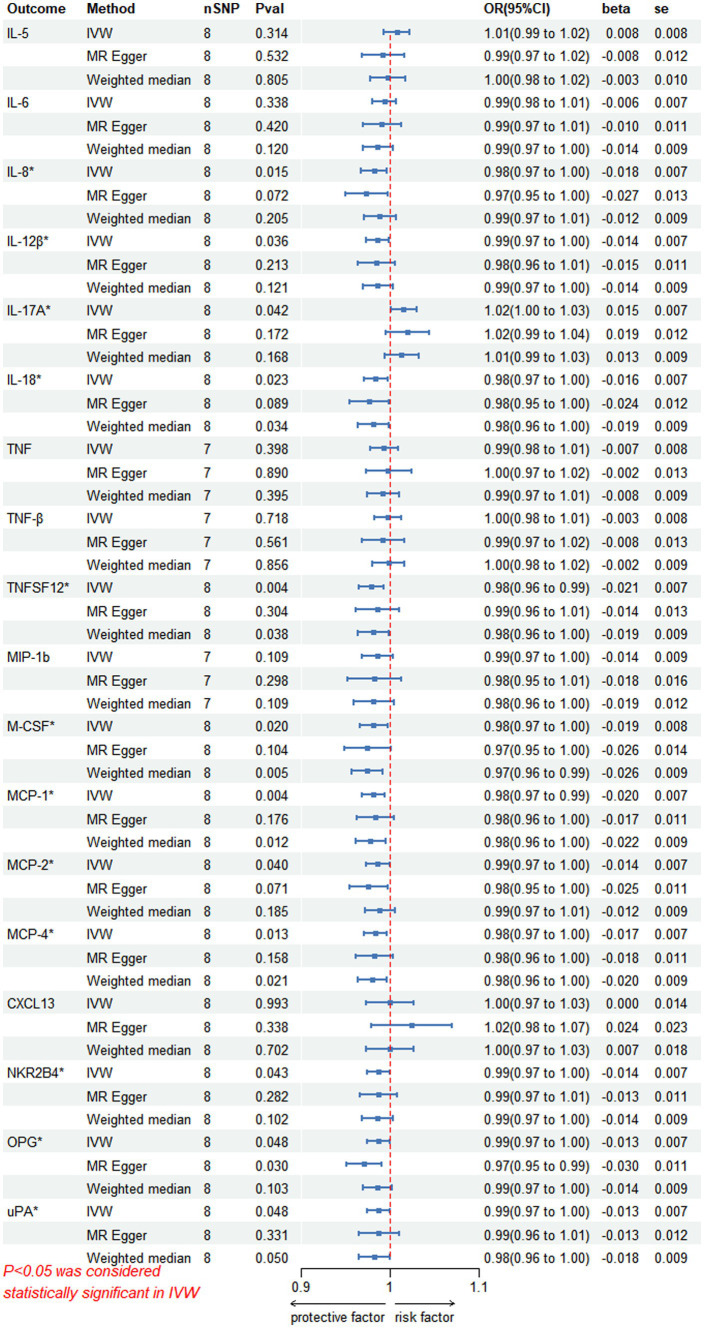
Reversed MR image showing PHN as the exposure agent and circulating inflammatory proteins as the outcome. The results of the IVW model demonstrated a significant correlation with a *p*-value lower than 0.05, consistent with genetic predictions; IVW, inverse variance weighted; OR, odds ratio; SNPs, single-nucleotide polymorphisms.

In the inverse MR analysis, we found that PHN was associated with 12 circulating inflammatory proteins, among which IL-8 (*p* = 0.015, OR 95%CI = 0.98 [0.97–1.00]), IL-12β (*p* = 0.036, OR 95%CI = 0.99 [0.97–1.00]), IL-18 (*p* = 0.023, OR 95%CI = 0.98[0.97–1.00]), TNFSF12 (*p* = 0.004, OR 95%CI = 0.98[0.96–0.99]), M-CSF (*p* = 0.020, OR 95%CI = 0.98 [0.97–1.00]), Monocyte chemotactic protein (MCP)-1 (*p* = 0.004, OR 95%CI = 0.98 [0.97–0.99]), MCP-2 (*p* = 0.040, OR 95%CI = 0.99 [0.97–1.00]), MCP-4 (*p* = 0.013, OR 95%CI = 0.98[0.97–1.00]), Natural killer cell R1-7 receptor2B4 (NKR2B4) (*p* = 0.043, OR 95%CI = 0.99[0.97–1.00]), Osteoprotegerin (OPG) (*p* = 0.048, OR 95%CI = 0.99[0.97–1.00]), urokinase-type plasminogen activator (uPA) (*p* = 0.048, OR 95%CI = 0.99[0.97–1.00]) had inverse causality with PHN, while IL-17A (*p* = 0.042, OR 95%CI = 1.02[1.00–1.03]) was positively associated with PHN; Bidirectional genetic causality only exists between IL-18 and PHN (Figure 6). And for the IL-18 dataset, we plotted the Manhattan plot after selecting SNPS with a threshold less than 5e-6 and removing the LD (Figures 3A,B). We also performed plotted a correlation heat map and forest plot to visualize our reverse study result (Figures 6, 3D).

**Figure 7:**
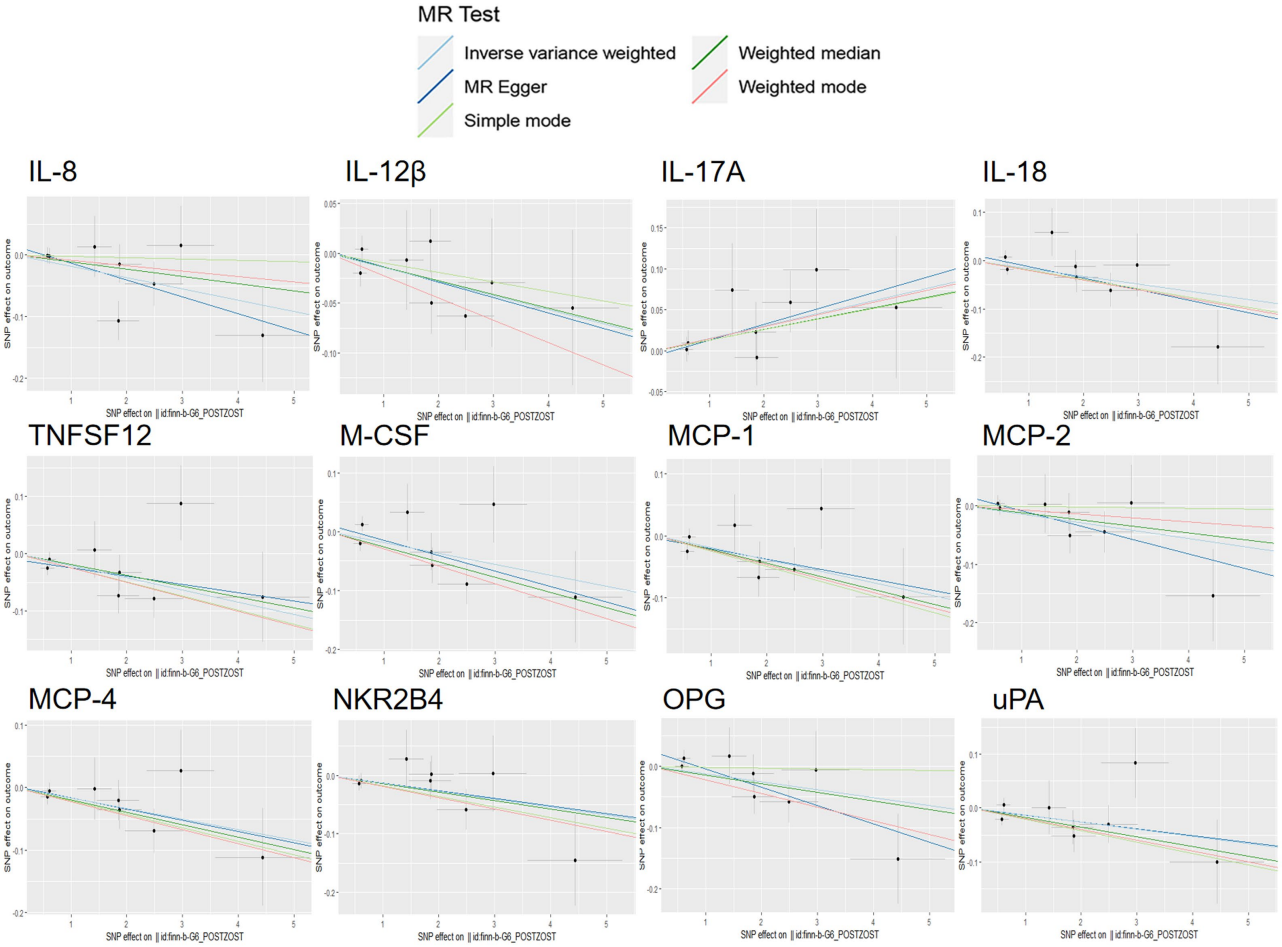
Scatter plots for the causal association between PHN and 12 circulating inflammatory proteins.

**Figure 8:**
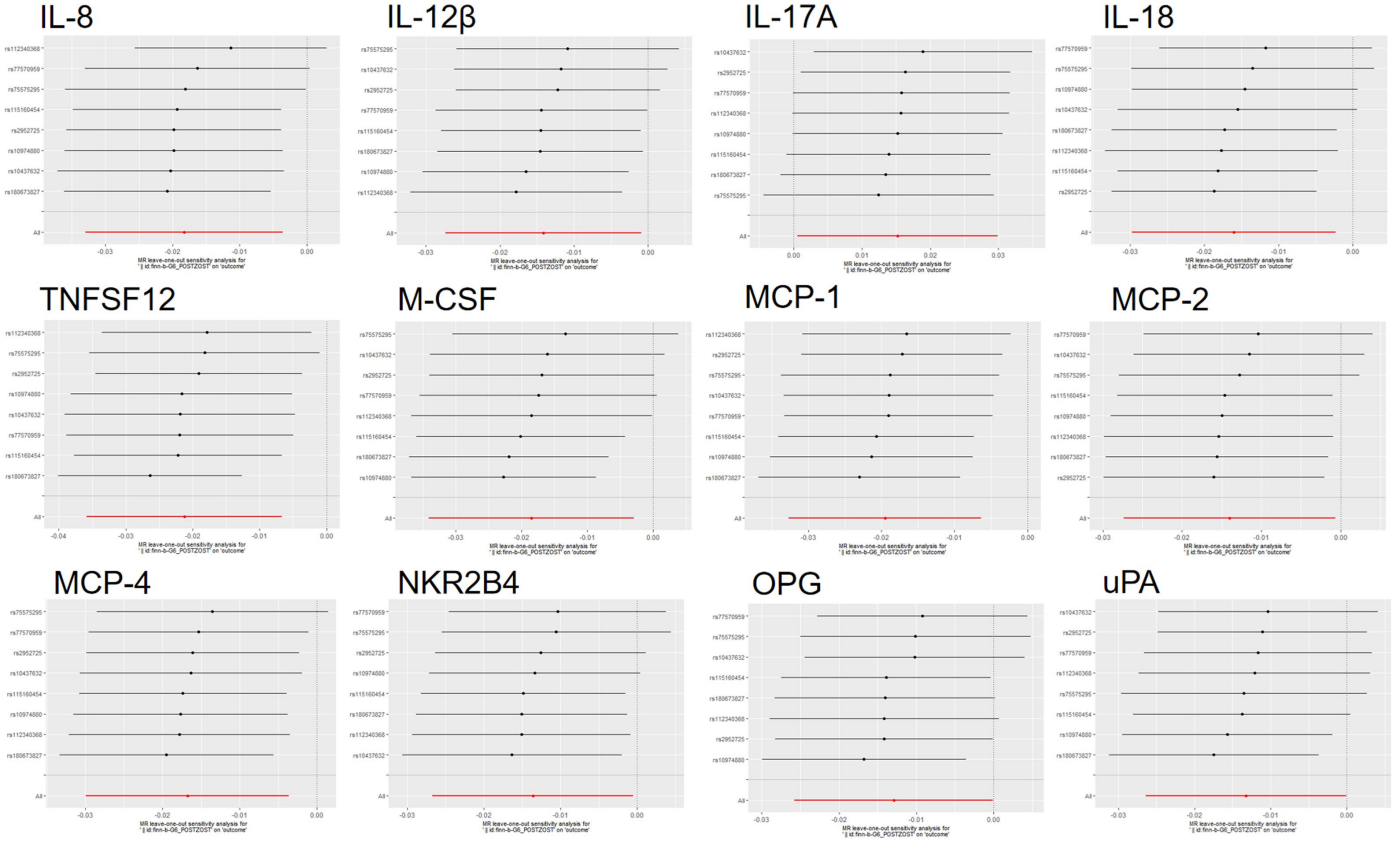
Reversed MR leave-one-out plot for the causal association between PHN and 12 circulating inflammatory proteins. The vertical axis shows the SNPs included in the analysis.

Scatterplot visualization and sensitivity analysis should also be demonstrated in the reverse study. Similar to the forward analyses, the scatter plots also showed consistent trends across the different MR tests, with IL-17A showing an upward trend (Figure 7). Leave-one-out sensitivity analyses were performed for each of the SNPs to determine the causal effect of PHN on the 12 inflammatory factors. We found that the result was always on one side of the zero line, regardless of whether any SNP was excluded (Figure 8). However, we did not detect any significant genetic variations between PHN and the other 82 circulating inflammatory proteins. The *p*-value of the MR-PRESSO global test and the p-value of the intercept from the MR Egger regression both indicate the absence of heterogeneity and pleiotropy (Table 2).

**Table 2:** Heterogeneity and pleiotropy analyses of the reverse MR data.

Outcome	Heterogeneity test				Pleiotropy test	
	IVW Q	*P*	MR-Egger *Q*	*P*	MR-Egger *P*	PRESSO *P*
IL-5	5.115	0.645	2.534	0.864	0.159	0.651
IL-6	4.33	0.741	4.203	0.649	0.733	0.757
IL-8	7.663	0.273	0.273	0.263	0.399	0.303
IL-12β	4.545	0.715	4.522	0.606	0.884	0.716
IL-17A	3.052	0.880	2.894	0.821	0.704	0.893
IL-18	7.557	0.373	6.756	0.343	0.431	0.432
TNF	6.618	0.469	5.816	0.444	0.404	0.958
TNFβ	8.75	0.271	8.430	0.208	0.649	0.99
TNFSF12	8.664	0.277	8.065	0.233	0.529	0.649
MIP-1b	6.012	0.421	5.890	0.316	0.760	0.5040
M-CSF	10.203	0.177	9.427	0.15	0.508	0.222
MCP-1	6.033	0.535	5.953	0.428	0.786	0.595
MCP-2	3.95	0.785	2.535	0.864	0.279	0.786
MCP-4	2.832	0.900	2.815	0.831	0.901	0.789
CXCL13	3.326	0.853	1.599	0.952	0.236	0.272
NKR2B4	4.269	0.748	4.266	0.64	0.954	0.750
OPG	7.307	0.397	3.257	0.775	0.090	0.415
uPA	7.027	0.426	7.022	0.318	0.950	0.492

## 4 Discussion

In this study, a bidirectional MR analysis was conducted to investigate the associations of circulating inflammatory proteins with PHN. The aim was to explore the genetic evidence of a potential causal relationship between circulating inflammatory cytokines and the risk of PHN. Our research revealed that IL-13, IL-18, MIP-1b, FGF-19, and SCF levels were linked to the likelihood of developing PHN according to forward analyses. Moreover, our reverse analyses showed that 12 circulating inflammatory proteins (IL-8, IL-12β, IL-17A, IL18, TNFSF12, M-CSF, MCP-1, MCP-2, MCP-4, NKR2B4, OPG, and uPA) had a genetic causal relationship with PHN, with IL-18 displaying a bidirectional causal link with this condition. In contrast, there was no significant correlation between PHN and the levels of inflammatory factors, including IL-4, IL-6, TNF-α, and CXCL13. This study offers epidemiological evidence supporting the use of pharmacological treatments that target inflammatory factors for the prevention and treatment of PHN.

PHN can persist for several months or even years, severely disrupting sleep and activities of daily living and causing significant physical, psychological, functional and social deficits. The pathogenesis of PHN is complex and not yet fully understood. However, an immune-mediated inflammatory response is believed to play a crucial role in its development. The inflammatory response involves a labyrinthine system of cells and mediators that includes cytokines, soluble receptors and other circulating proteins. Numerous inflammatory cytokines, including TNF-α and interleukins (IL-1 and IL-8), have been demonstrated to have essential functions in neuralgia generation (43–45). Previous MR studies have demonstrated a correlation between chronic inflammation of the gastrointestinal tract and infection with the VZV. This suggests that VZV infection is causally associated with inflammatory Crohn’s disease. Furthermore, the microenvironment infiltrated with inflammatory proteins is thought to promote VZV infection (46). The first MR study on inflammatory factors in PHN investigated the potential causal relationship between IL-18 and PHN (36). The findings indicated that IL-18 may act as a risk factor for PHN, exerting its pain-promoting effects by modulating pain signaling pathways in the spinal cord and brain. As the second MR study of inflammatory proteins and PHN, this study incorporates a larger dataset of inflammatory factors and performs bidirectional validation, thereby complementing the first study and fleshing out the pathogenesis of PHN.

IL-18, which is an interleukin, is a cytokine member of the IL-1 superfamily (47). It is produced by dendritic cells, macrophages and monocytes and possesses a structure similar to that of IL-1β and stimulates the secretion of interferon (IFN) gamma by natural killer (NK) cells (48). It has a broad immunomodulatory function and plays a crucial role as a regulatory proinflammatory cytokine in the immune response and inflammation (49). It plays an important role in the regulation of glial cell activity in the dorsal root ganglion and promotes the interaction of microglia and astrocytes in the monocyte–macrophage system to regulate neuronal activity, thus promoting the development of inflammation and neuropathic pain-touch hypersensitivity symptoms (50–52). Relevant animal studies have shown that blocking IL-18 with an endogenous inhibitor of IL-18 (IL-18 binding protein, IL-18BP) reduces pain sensitivity and ameliorates cold nociceptive hypersensitivity and tactile anomalies in pain (53). In this study, we discovered for the first time that IL-13, an anti-inflammatory cytokine, is strongly linked to PHN. It can trigger NK cells to generate IFN, which then boosts the activation of monocyte-macrophages and the cellular immune response, thereby opening up fresh perspectives on PHN pathogenesis (54, 55).

Stem cells can self-replicate and undergo diverse differentiation mechanisms, making them potentially useful for alleviating neuropathic pain through their ability to differentiate into neuronal and glial cells, release neurological nutrients for nerve repair and regeneration, and inhibit inflammation. Several clinical trials have demonstrated the efficacy of stem cell transplantation in improving nerve pain symptoms among patients. As such, stem cell therapy has emerged as a viable alternative for treating nerve pain (56, 57). SCF is an acidic glycoprotein that stromal cells produce in the bone marrow microenvironment. Its main impact is on early pluripotent stem cells, and it has strong synergistic effects on other cytokines. Our study identified stem cell growth factor as a protective factor in PHN, and may pave the way for new therapeutic approaches, such as combining SCF with stem cell transplantation therapy, to improve the efficacy of PHN treatment (58, 59). Furthermore, certain studies have shown that within the epidermal layer of herpes zoster lesions, epidermal keratinocytes exhibit high expression of MIP-1 following viral infection. Alternatively, these keratinocytes may induce the secretion of MCP-2 from human monocytes via TLR1 or through interactions with fibroblasts (60–62). The results of the positive analyses in this study indicate that MIP-1b and FGF-19 are risk factors for PHN, providing a basis for genetic support.

Currently, drug therapy and minimally invasive interventions are the mainstays of clinical treatment for PHN. Drug therapy options include nutritive nerve agents, such as vitamin B1 and methylcobalamin tablets, as well as antiepileptic medications, such as pregabalin, gabapentin, and the tricyclic antidepressant amitriptyline. Interventional approaches include nerve block, neuromodulating pulsed radiofrequency, and electric stimulation of the spinal cord, among others (1, 63–66). However, it is important to note that there is no universally effective method for treating all patients with PHN. Moreover, drug treatments can cause side effects, including nausea, vomiting, and dizziness. The prevalence of PHN is on the rise, posing a significant chronic pain dilemma requiring immediate attention. A decrease in quality of life and an increase in healthcare expenses can be attributed to this type of NP. Therefore, there is still a need to investigate the pathogenesis of PHN to advance the study of relevant targeted therapeutic agents and treatment protocols with greater efficacy.

MR analyses could be used to explore the causal relationship between circulating inflammatory proteins and PHN. Compared with previous analyses, this study has greater robustness in excluding confounding factors. Genetic variations in 91 of these circulating inflammatory proteins were acquired from the most recent GWAS analysis available in August 2023, safeguarding the robustness of the instrumental factors in the MR analyses. Additionally, the use of nonoverlapping exposures and pooled data for the outcomes proved effective in preventing bias. This study has certain limitations, as the sample size was small, which resulted in most SNPs used in the analysis not meeting the traditional GWAS significance threshold (*p* < 5 × 10–8) and instead being analyzed using a threshold of *p* < 5 × 10–6. Therefore, it is necessary to conduct two-way analyses when the sample size of the data being analyzed increases. Second, the majority of the aggregated data in this research pertains to individuals of European descent; hence, it is necessary to authenticate the relevance of our discoveries to other populations through the use of local GWAS data.

## 5 Conclusion

This MR study revealed that various circulating inflammatory factors, such as IL-18, are causally linked with PHN at the genetic level and that this association shows bidirectional genetic causality with PHN. The outcomes of several of the previously conducted studies have been verified, but further randomized controlled trials (RCTs) are needed to determine the specific mechanisms of the various newly discovered inflammatory factors associated with PHN. The other inflammatory proteins, such as TNF-α and IL-6, are not genetically causally linked to PHN. Nevertheless, this does not preclude the likelihood of their linkage at nongenetic levels. This research makes a significant contribution to understanding particular inflammatory biomarker pathways involved in the pathogenesis of PHN. The findings of this study could lead to the development of innovative preventative and targeted treatments for PHN.

## Data availability statement

The original contributions presented in the study are included in the article/supplementary material, further inquiries can be directed to the corresponding authors.

## Author contributions

WL: Data curation, Methodology, Software, Visualization, Writing – original draft, Writing – review & editing. HH: Conceptualization, Investigation, Visualization, Writing – original draft. CL: Conceptualization, Formal analysis, Methodology, Project administration, Writing – review & editing. YL: Formal analysis, Methodology, Visualization, Writing – review & editing. PM: Funding acquisition, Project administration, Resources, Investigation, Supervision, Writing – review & editing. BF: Funding acquisition, Project administration, Resources, Supervision, Validation, Writing – original draft, Writing – review & editing.

## Footnotes

^1^ http://www.phenoscanner.medschl.cam.ac.uk/phenoscanner
